# Dynamic Gait Index - Brazilian Version

**DOI:** 10.1016/S1808-8694(15)31050-8

**Published:** 2015-10-19

**Authors:** Sandra Meirelles De Castro, Monica Rodrigues Perracini, Fernando Freitas Ganança

**Affiliations:** 1 Physical therapist. Post-Graduate student in Neuromotor Rehabilitation Sciences - UNIBAN.; 2 Physical therapist. PhD in Rehabilitation Sciences - UNIFESP - EPM. Professor and M.S. coordinator in Physical Therapy - Cidade de São Paulo University.; 3 Otorhinolaryngologist; PhD in Medicine - UNIFESP - EPM, Associate Professor of Neuro-otology - UNIFESP - EPM. Postgraduation Course Professor of Neuromotor Rehabilitation Sciences - UNIBAN. Universidade Bandeirante de São Paulo - Vestibular Rehabilitation Department - Division of Neuro-otology - UNIFESP - EPM.

**Keywords:** equilibrium, rehabilitation outcome assessment, reproducibility of results, dizziness

## Abstract

The Dynamic Gait Index (DGI) is a useful test to evaluate balance and gait. **Aims:** The objectives of this study were to culturally adjust the DGI to the Portuguese language and to assess its reliability. **Methods:** The method proposed by Guillemin et al. (1993) was used for a cultural adaptation of this tool. A prospective study was performed with 46 patients that were assessed in the cultural adaptation phase. The items that not understood by 20% or more patients were reworded and reapplied. The final Portuguese version of DGI was applied to 35 elderly in order to check intra and interobserver reliability. The Spearman rank coefficient was used to correlate intra and interobserver scores and the Wilcoxon test was applied to compare these scores. Internal consistency was analyzed by the Cronbach alpha coefficient. **Results:** There were statistically significant correlations among the scores for intra and interobserver assessments for all items (p<0.001), which were classified as good and very strong correlations (ranging from r=0.655 to r=0.951). The DGI demonstrated high internal consistency in intra and interobserver assessments (varying from ∝=0.820 to ∝=0.894). **Conclusion:** The DGI was culturally adjusted to Brazilian Portuguese and proved to be a reliable tool.

## INTRODUCTION

Brazil is going through an important demographic transition, with a matching increase in its elderly population. This demographic trend is being followed by an epidemiological transition, with a change in the population morbi-mortality profile. Currently, the major causes of death in our country are chronic diseases. Therefore, the trend is to have a growing number of old people, that, although they live longer, they will face social, psychological and physical deficits, thus increasing their demand for specialized care^1^, ^2^, ^3^.

The elderly, especially the ones with the most advanced ages, bear coexisting diseases, most of these are chronic-degenerative, that if not duly treated, tend to present complications and sequels, and this may cause a reduction in functional capacity, affecting both the independence and the autonomy of this population^4^, ^5^, ^6^.

Among the most common complaints of the elderly we have: body balance disorders - clinically characterized as vertigo and other types of dizziness, lack of proper body balance, gait deviation, nausea, instability and falls. Balance disorders account for one of the most important etiological factors related to falls and unsteadiness in the elderly, being a marker of frailty and prone to cause functional incapacity and dependence^7^, ^8^, ^9^.

Balance loss prevention and rehabilitation in the elderly require the development of proper clinical research protocols to assess and measure balance, falling risk and the very mobility of this population, as well as establishing rehabilitation programs. There are a number of functional assessment tools used to identify balance and mobility problems, and the observer has to choose the type of approach that better serves the investigation goals.

Such tools are widely used in the clinical practice and in scientific research, since they assess the individual’s performance in complex tasks, based on basic and instrumental daily life activities, as well as balance and mobility characteristics^10^, ^11^. Most of these tools were created in another language. Therefore, the professional who investigates functional alterations in the elderly is challenged for not having such tools adapted to our culture, and that are reliable, accurate and sensitive in the identification and measurement of these aspects^12^, ^13^, ^14^.

**Chart 1:**
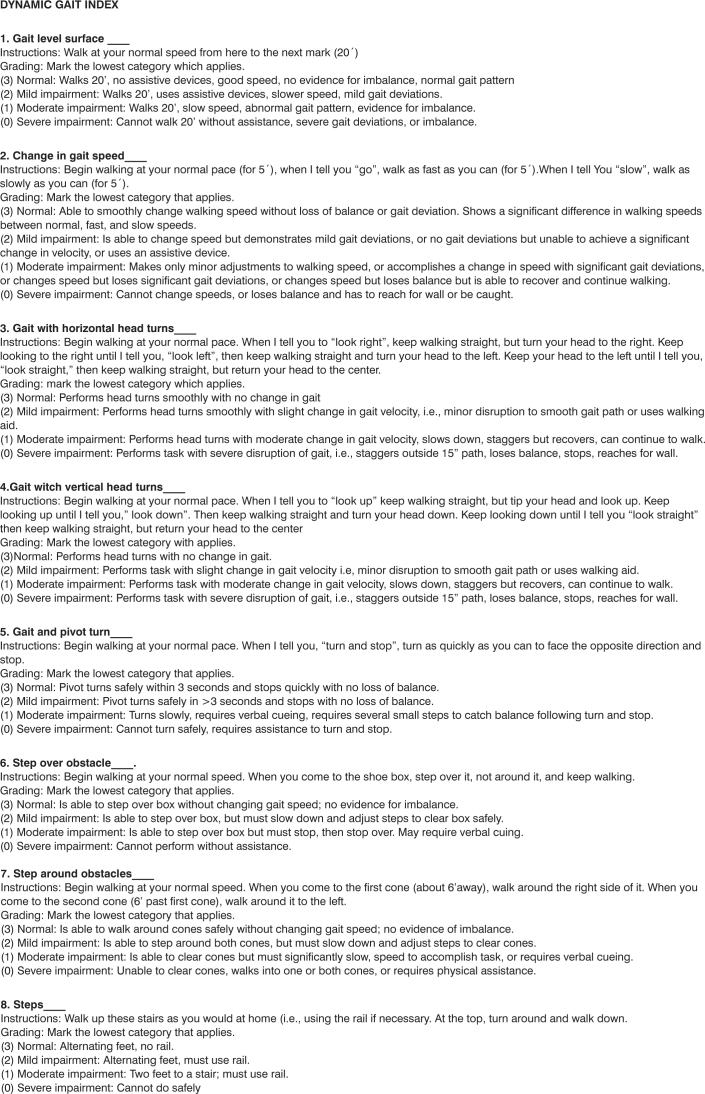
DGI in its original English language

Shumway-Cook et al.^15^ developed a functional mobility assessment tool, the Dynamic Gait Index (DGI), with the goal of assessing and documenting the patient’s capacity to change his/her gait in response to changes in the demands of certain tasks, in the elderly with balance impairment. DGI is made up of eight tasks involving gait in different sensorial contexts, including gait on a flat surface; gait speed changes; horizontal and vertical head movements; going over and around obstacles; turn around on one’s own body axis, going up and down steps^15^ (Chart 1).

The original DGI version was created in English, and in order for it to be properly used in our settings, it has to go through a cultural adaptation, followed by its reliability assessment.

Our goals with the present study is to make a cultural adaptation of the DGI for Brazilian Portuguese and assess its reliability.

## METHODS

This study was carried out within the vestibular rehabilitation research line of the Post-Graduation Program in Neuromotor Rehabilitation of the Universidade Bandeirante de São Paulo, approved by the ethics committee of this institution under protocol # 016/2004.

We included 71 elderly patients referred from the Geriatrics and Neuro-otology outpatient wards of the UNIFESP/EPM, with 65 years or older, from both genders, who underwent DGI testing. The sample underwent a cognition evaluation through the mini-mental test16 (MEEM). All the patients agreed to participate in the research, and signed freely an informed consent form.

We followed the method developed by Guillemin et al. (1993) in order to perform the DGI cultural adaptation. The latter was individually translated by three English teachers, who are fluent in English and aware of our goal with such study. From the original English version we then had three initial translations into Portuguese.

The revising committee was made up of two physical therapists and one physician, all Brazilians and fluent in the English Language. This committee of specialists then produced the first version of this tool in Brazilian Portuguese, based upon the three initial translations, comparing them, reducing the differences, choosing the best words and expressions for our Brazilian population, preserving the original concepts of the tool.

This first Brazilian Portuguese version was then submitted to two other translations into English - Back-Translation - carried out independently by two bilingual translators, native speakers of English, who did not know each other and did not know of the original version in English, nor about the goals of the present study.

The two English versions where then submitted to the evaluation of the same revising committee, who compared them to the original version in English in order to detect possible translation errors. The committee then discussed the items present in the initial Brazilian Portuguese version, the differences were analyzed and replaced by suitable words or expressions, so that the second version in Portuguese would be clear and equivalent to the English original text.

The Portuguese version was deployed as many times as it was necessary until all the items were well understood by the patients in order to establish cultural equivalence.

In order to assess the level of understanding by the patients, we added a questionnaire to the tool. After reading the instructions, the interviewer was required to classify the patients’ understanding level in 4 categories: 1 “Understands”; 2 “Requires repeating the command or part of it”; 3 “Requires demonstrating the task, or part of it”; 4 “Does not understand”. The latter was considered when any doubt regarding the given verbal command arose, and the data were noted. That item considered as not understood was read again, if necessary, the words or expressions were clarified, until the patient fully understood the instruction. In cases in which after the item was read and explained the patient remained doubtful, the task was demonstrated by the investigator, and then performed by the patient. The issues that were difficult for 20% or more patients were reformulated.

In order to test inter and intra-observer reliability of the final Brazilian version of the DGI, the tool was used three times in the same sample of elderly patients. The first two assessments were applied on the same day, by two observers (Obs 1 and Obs 2), for the inter-observers evaluation, with a 30 minute interval between the first and the second application, and the first interview with the tool for each patient was carried out alternating the order of interviewers.

The third assessment was carried out 7 days later by observer 1 (Obs 1 re-testing), for intra-observer assessment, after investigating changes in the patients’ general status and medication during this period. Those patients that showed changes in their general status or in medication used that could alter task performance were excluded from the study.

For cultural adaptation purposes, the tool was applied to 46 elderly patients; and in the intra and inter-observers reliability study we assessed 35 patients, 10 of these individuals also participated in the cultural adaptation of the program.

In order to deploy the final DGI version we used two rubber traffic cone obstacles of 0.50cm height and 1 shoe box with 40cm of length, 20cm in width and 15cm in height.

Each patient was assessed by means of an ordinal scale with 4 categories and scored according to his/her own performance in each task: 3 = normal gait, 2 = slight impairment, 1 = moderate impairment and 0 = severe impairment. Maximum scoring is 24 points, and a score of 19 points or less is able to predict a risk of falls15,17.

In order to analyze the correlation of intra and inter-observer scores, we also used the Spearman correlation coefficient. In order to compare inter and intra-observer scores we used the Wilcoxon test for paired or relational data.

In order to assess the inner uniformity of the final Brazilian version of the DGI, we used the Cronbach alpha coefficient. Values above 0.70 indicate high uniformity. We used the Spearman correlation coefficient in order to correlate each one of the 8 items with the total DGI score, according to scores given by Obs 1, Obs 2 and Obs 1 re-testing.

Significance level used for statistical analysis was of 5% (p<0.05).

For the whole statistical analysis we used the SAS (Statistical Analysis System) for Windows, version 6.12 software.

## RESULTS

The DGI original version generated three translations of this tool into Portuguese that have contributed to formulating the final DGI Brazilian version by the revising committee.

The English translation (back-translation) of the initial DGI Portuguese version produced 2 documents which were equivalent to the original DGI version and from these versions a second DGI version into Portuguese was created.

Items 1, 2, 3, 4, 6 and 8 were understood by more than 80% of the population assessed; notwithstanding, the instructions on items 3 and 4 were reformulated by the revising committee, since they had long phrases and with repetitive words or expressions, the latter were deleted, thus making the text more objective and with shorter phrases. Items 5 and 7 presented many interpretation problems; difficulties were recorded and the instructions were discussed and changed by the committee of specialists.

After the necessary changes needed to improve the understanding of the items, the third version was created and used on 10 other patients. Items 3 and 4 did not present understanding problems; however, items 5 and 7 were again considered difficult for more than 20% of the patients. Item number 5 (gait and pivoting over one’s own body axis) had the following instructions on its third version: “Start walking as you normally do. When I say turn and stop, turn as quickly as possible to the opposite direction and stop”, led to many doubts such as “In what direction should I stop? Should I turn forward?” or the patient would turn only the torso, without lifting the feet from the floor or he would do a whole turn to one of the sides and stop. The expression “Turn to the opposite direction” was not well understood” and the item had to be modified by adding words that could better explain the task. The instruction was then substituted for: “Start walking as you normally do, when I say turn and stop, turn backwards towards your starting point and stop, as quickly as possible”. Again, the same doubts arose, and after the item was thoroughly analyzed and the questions presented by the patients, the committee decided to add expressions and verbal tips to the text, such as “this point”, in order to indicate the direction the patient should turn and stop, aiming at making the instruction as clear as possible. The instructions were replaced by “Start walking as you normally do. When I say turn and stop, turn as quickly as possible towards the opposite direction and stand still facing (this point), your starting point (Fourth Brazilian version) “.

Item 7 instructions (going around obstacles): “Start walking as you normally do. When you get to the first cone (located at about 1.8 meter), go around it by the right side. When you get to the second cone (located about 1.8m after the first one), go around it by the left side”- presented may interpretation challenges, when the patients had doubts as to side, since most went around the cones using the side other than the one requested. In order to solve the problem, we added a new command to the instruction, such as “pass between the cones and keep walking”. The expression “going around” caused interpretation errors, and the patients circled each one of the two cones, without going between them.

The revising committee decided to add the expression “go around the cones” to item 7 in the first phrase, so that the patient would understand the task goal right at the beginning of the instructions, avoiding confusion and forgetting important data. As to the mistake of changing the sides in which to go around the cones, we decided not to change the text, since this fact does not alter the task goal and also does not interfere in the patient’s performance. The item was changed into: “Start walking as you normally do, and go around the cones. When you reach the first cone (at about 1.8m), go around it through the right side, go between them, keep walking, and when you reach the second cone (at about 1.8m after the first one), go around it through the left side “.

**Chart 2:**
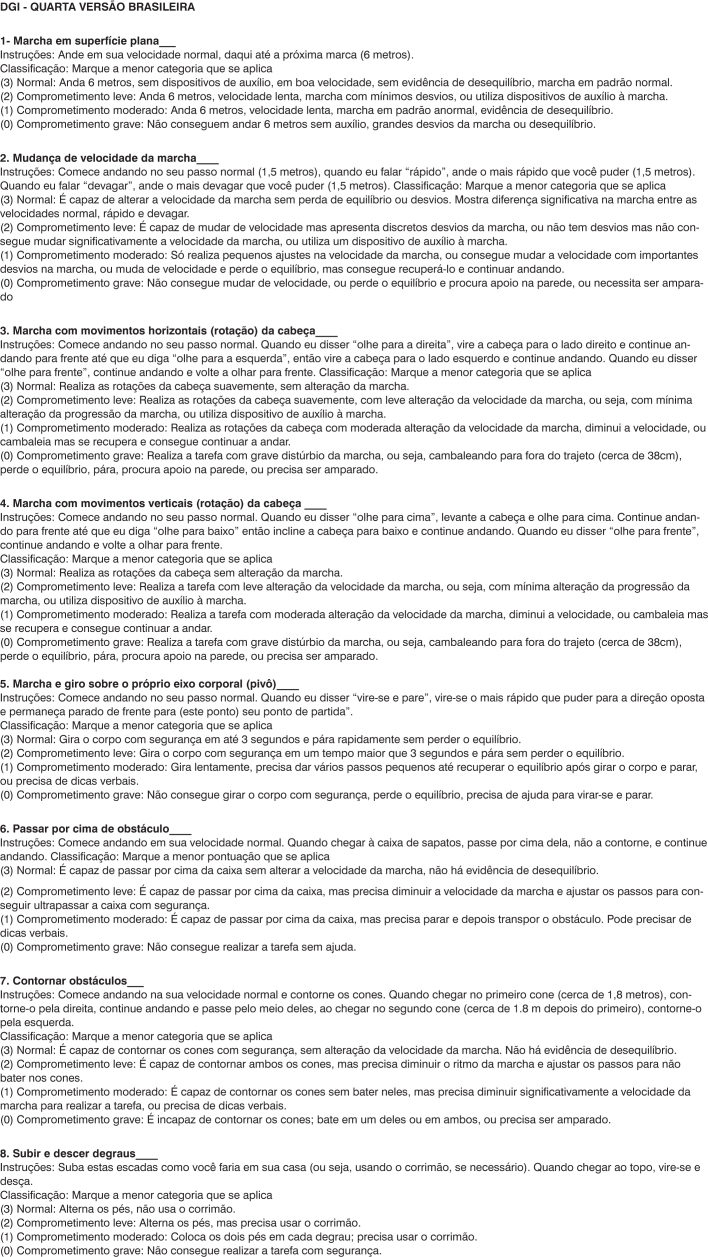
Final Brazilian Version of the DGI

A fourth and last version of the Brazilian DGI (Chart 2) was created by the revising committee, who replaced some words by others with the same meaning, and added new words and expressions in order to better detail the commands. The fourth version was then used in six other elderly patients, and this time, items 5 and 7 were understood by 100% of the patients, thus making this the final Portuguese version of the DGI.

There was a statistically significant correlation between inter-observer scores for all the items and also for the tool total score (p<0.001). The values obtained for correlation analysis for the items individually varied from r=0.655 to r=0.951, indicating that the correlation between inter-observer scores was good and strong. We obtained a strong inter-observers correlation for the DGI total score (r=0.893).

When we compared the scores between observers 1 and 2, there was a statistically significant difference in score only for item (Z=-2.12; p=0.034), indicating higher scores for Obs 2. For the remaining items and the total score there was no inter-observer significant difference. Values obtained for individual items varied from Z=-2.12; p=0.034 to Z=0.58; p=0.564; and it was of Z=-0.62; p=0.535 for total DGI.

There was a statistically significant correlation between intra-observer scores for all the items, and also for the total DGI score (p<0.001). The values obtained through the correlation analysis for the individual items varied from r=0.646 to r=0.930, indicating that the intra-observer correlation varied from good to very strong. We obtained a very strong intra-observer correlation for the total DGI score (r=0.919).

When we compared the intra-observer correlation, there was a statistically significant difference in scoring between test and re-testing (Obs1) for items 3 and 6 and for total DGI score, with values of Z=-2.33; p=0.020, Z=-2.24; p=0.025 e Z=-2.06; p=0.040, respectively. These values were higher in the re-testing. For the other items present in the DGI, there was no significant intra-observer difference. The values obtained for the items individually varied from Z=-2.3; p=0.20 to=0.8; p=0.564.

There was a high internal uniformity of the DGI in the final Brazilian Portuguese version for Obs 1, Obs 2 and Obs 1 re-testing with values of a=0.847; a=0.820 and a=0.894, respectively. Item 5 was the one with the least correlation with total DGI.

We noticed a significant correlation for all the items (p=0.0001), except item 5, with a weak correlation for those values from Obs 1 (r=0.312; p=0.672) and Obs 2 (r=0.435; p=0.008). The correlation of the remaining items with the total score varied for Obs 1 from good to strong (r=0.649 to r=0.859), for Obs 2 the correlation went from moderate to strong (r=0.506 to r=0.841) and for Obs 1 re-testing the correlation varied from good to strong (r=0.637 to r=0.836).

Average age of the patients assessed was of 75.54 ± 6.99 years, varying between 65 and 91 years, and most of the patients were in the age range between 70 and 74 years (28.1%). As to cognitive assessment, most patients scored above the cutting point (24 points) in the Mini-Mental exam (85.9%), with average score of 26.31± 2.73.

## DISCUSSION

On the cultural equivalence phase, items 5 and 7 presented several understanding problems in their instructions.

Instructions on item 5: “start walking as you normally do, when I say turn and stop, turn as fast as you can to the opposite direction and stop “, stirred the most frequent doubts on patients, regarding the expressions “turn” and “opposite direction”. Many patients turned their bodies on one direction, or turned only their torsos on the opposite direction, without moving their feet. All the patients who had difficulties understanding the item were able to understand it after the command was repeated, or when the task was demonstrated, as a last resort. The item was well understood after having been modified, when the patients were manually instructed as to the point at which they should turn.

Item 7 brought about some interpretation and understanding hurdles. The task major goal is to go around obstacles made up of two traffic cones, at about 1.8m away from each other. Many patients had difficulties as to which would be the correct side to go around the cone; another common doubt was in relation to the expression “go around”, that some understood that they had to do a complete turn around the cone. For instructions to become clearer and more easily understood, we added to the text the expression “go in between them” and “then go around them” in order to provide the patients with more details regarding the task.

It was broadly acknowledged that it is hard for the patient to understand all the instructions they should follow in a more complex text if they only read about them once. To provide verbal clues or body demonstrations would be interesting, because it would enhance understanding and facilitate the interpretation of such instructions. Such fact was seen when the item was repeated and clues were provided, or when the task was demonstrated, in which the instructions were correctly executed.

The evaluators also had their doubts in relation to the point assigned between one score and another, because items 5 and 6 had similarities in their performance description or because there was no description of the skill presented during task execution.

During item 5, many patients, when they turned their bodies quickly in less than 3.1 seconds, they were somewhat unsteady after they stopped. Notwithstanding, there was a grading option for this result. If the observer does not pay attention, or does not consider the instability presented by the patients and only concerns him/herself with the time, he/she may overestimate the score and thus, compromise the final result.

During item 6, many patients presented with postural unsteadiness after going by the box, however they recovered and kept on walking. Another alteration presented by patients was that they hit and stumbled upon the box, with and without balance; however, they all recovered and kept on walking. Notwithstanding, there is no score describing these alterations. Thus, we advise people that when they use DGI, the specialist should be familiarized with the tool, and all the assessment possibilities must be be predicted and standardized.

Methodological criteria used in this paper were rigorously followed. During the translation process, it was possible to observe that the use of such criteria makes the translation easier and it is also easier to adapt the tool to our Portuguese language. Concurrently, its conceptual equivalence may be obtained by means of a consensus, with the help from specialists and patients stating their difficulties and opinions. Patient participation in this process increased its possibility of adaptation to the target population.

The lack of a proper translation and cultural adaptation method may introduce a number of biases and problems. The researchers have to report on all the stages of the translation, detail the results of test application on patients, as well as the details of the cultural adaptation and, mainly, concern themselves with the psychometric properties, such as the intra and inter-observer reliability, and also the tool internal consistency^12^, ^14^.

Comparing the values of the inter-observer scores we noticed a significant difference among them for item 6, with higher scores for Obs 2. Such differences may have occurred because of point assignment criteria of one or another score in case the patient’s performance is not characterized in any of the options, or in cases in which the options had very similar descriptions, creating doubts at the time of scoring. In this case, evaluation criteria for Obs 1 were more strict. It would have been better if these evaluation options had been considered and standardized prior to the study.

Our intra and inter-observer reliability was different from that obtained by Shumway-Cook et al.^11^ (0.96 and 0.98), Wrisley et al.^18^ (0.95), McConvey et al.^19^ (0.93 and 0.98), respectively. The scoring carried out by these authors, simultaneously in the same patient, besides eliminating performance variability detected at different assessment moments in the same subject, may explain the higher reliability values found in their studies. We have to take into account that DGI tasks evaluate skills in different contexts with dynamic characteristics, and each patient’s performance may vary with each assessment. Items 3, 4 and 5 may have more complex tasks, and are considered more difficult to perform, such as tasks in which the patient has to rotate the head while walking and turn quickly, and they may present performance variations in subsequent tests.

Other relevant points, as far as scoring is concerned, are the lack of operational instructions in order to use the tool and assign points to the scores, lack of materials, measures and environment standardization. One good example is the very size of the shoe box; the larger the box, the greater is the difficulty in executing the task. Other examples are variations in room size, lighting and noise conditions, as well as differences in floor and stairs characteristics.

DGI has a simple format, with scoring definitions according to performance in each task. However, additional instructions as to deployment criteria or for scoring decision making were not published. The scores have performance descriptions, than for most items is made of adjectives. In our opinion, such definitions become subjective when doubts arise during scoring. Both reliability and accuracy of this tool could increase if the items had quantitative measures, besides instructions deployment and better defined criteria to grade each item. We suggest a next step in this study, which is to rethink response options and deployment standardization.

We noticed a high internal consistence for this tool. Item 5 was the one with the least internal consistency; however, did not prove to have a significant bias, thus keeping the tool stable. When we relate internal consistency data to the data observed in the current paper, most patients scored high in item # 5, for both Obs 1 and Obs 2 and in the re-testing. Data showed a ceiling effect for this item. The task is basically to turn one’s body in less than 3.1 seconds and one limitation was that we did not measure time in a watch or stopwatch, and that could have caused punctuation errors; another important observation, already mentioned in this discussion, was the lack of a grading option for those patients that turned their bodies in less than 3.1 seconds, with evidences of unsteadiness.

In the international literature there are DGI publications with reliability studies; however, without the methodological criteria of the present study^11^, ^18^, ^19^. It is well known that a measurement tool is very useful when it bears accuracy and reliability, and a proper internal consistency is a reliability indicator of the measure. Intra and inter-observer reliability are extremely important properties in evaluations that measure skills, especially tools that bear many items, which are clinically summarized by means of joining scores^20^.

As to sample characteristics, we chose to include elderly citizens aged 65 and above coming from the neuro-otology or geriatric wards, because the original version of this tool was created to assess the elderly with balance problems.

It is our opinion that most of the elderly included in this study were functionally independent, made their own decisions, walked and used public transportation in order to attend the scheduled appointments, and had no evidence of cognitive impairment. Low MEEM scores presented by some patients may be explained by the low educational level of these patients. In the present investigation, results attained in the MEEM (averaging 26.31) were no different from those obtained in studies carried out with elderly with low educational level^21^, ^22^.

DGI cultural adaptation and its reliability assessment carried out in the present investigation may contribute to the Brazilian scientific community, for its use in clinical practice, and also for future research projects involving body balance and mobility.

## CONCLUSION

DGI has been culturally adapted into Brazilian Portuguese and proved to be a reliable tool.
